# Ayurveda and Urinary Tract Infections

**DOI:** 10.4103/0975-1483.66811

**Published:** 2010

**Authors:** R Jain, S Kosta, A Tiwari

**Affiliations:** *School of Biotechnology, Rajiv Gandhi Proudyogiki Vishwavidyalaya, Airport Bypass Road, Gandhi Nagar, Bhopal (M.P.) - 462 036, India*

Sir,

Urinary tract infections (UTIs), the second most common type of infection in the body is one the most serious health problem affecting millions of people each year. The urinary tract infection (UTI) involves infection in the kidneys, ureters, bladder or urethra. These are the organs that urine passes through when eliminated from the body. Women are especially prone to UTIs, even though they generally have anatomically and physiologically normal urinary tract.[1] Reasons for this are not yet well understood. These infections can be very serious when they do occur in men; however, UTIs in men are not as common as in women. A urinary tract infection (UTI) also known as Fearnes syndrome is a microbial infection that affects any part of the urinary tract. Normally, urine is sterile. It is usually free of microbes but does contain fluids, salts and waste products. The main cause agent in at least 90% of uncomplicated infections is *Escherichia coli*, which live in the bowel (colon) and around the anus. An infection occurs when bacteria get into the bladder or kidney and begin to grow. The infection often starts at the opening of the urethra where the urine leaves the body and moves upward into the urinary tract. Abnormalities of the urinary tract that hinder the flow of urine set the stage for an infection.

Although modern antibiotics are being used in UTIs, urinary tract infections can be quickly and easily treated with a herbal treatment with no side effects. Herbs known for the management of urinary tract Infections and other urinary disorders divided in important categories: (a) Urinary antiseptic and anti-adhesion herbs like *Juniperus* spp., *Vaccinium macrocarpon, Salvia officinalis, Punica granatum, Tribulus terrestris, Terminalia chebula, Ocimum sanctum, Cinnamomum cassia, Azadirachta indica* and *Ocimum sanctum*,[2,3] which are effective against major urinary tract pathogens namely *E. coli, Klebsiella pneumoniae, Pseudomonas aeruginosa* and *Enterococcus faecalis* (d) Bladder protectives that control bladder and protect from infections comprising of *Equisetum arvense, Hydrangea petiolaris* and *Zea mays*[4] (e) Kidney care for instance, *Boerhaavia diffusa, Eupatorium purpureum, Agropyron repens* and *Berberis vulgaris* and (f) Herbs for symptoms of benign prostatic hyperplasia, most notably *Serenoa repens* and *Prunus africana*. All these herbs are discerned to know different type of phytoconstituents and show potential in the treatment of urinary disorders and could be alternative to uropathogen resistance to the antibiotic during a UTI.
